# Understanding the patient and family experience of nutrition and dietetic support during childhood cancer treatment

**DOI:** 10.1007/s00520-023-07787-3

**Published:** 2023-05-08

**Authors:** Emma Clarke, Gemma Pugh, Eveline van den Heuvel, Mark Winstanley, Andrew C. Wood, Stephen J. Laughton, Amy L. Lovell

**Affiliations:** 1 The University of Auckland, Private Bag 92019, Auckland, 1142 New Zealand; 2 National Child Cancer Network, Te Aho O Te Kahu Cancer Control Agency, Wellington, New Zealand; 3 Starship Blood and Cancer Centre, Te Whatu Ora, Te Toka Tumai, Auckland, New Zealand

**Keywords:** Childhood cancer, Patient perspectives, Nutrition support

## Abstract

**Purpose:**

This study aimed to understand the experience of families caring for a child with cancer in New Zealand (NZ) who received nutrition and dietetic support during cancer treatment and their preferences for the delivery, format, and timing of nutrition information.

**Methods:**

Childhood cancer patients and their families (*N* = 21) participated in a mixed-methods study at a specialist paediatric oncology centre in Auckland, NZ. Before the semi-structured interview, participants completed a questionnaire capturing demographic, disease, and treatment characteristics of their child, their nutrition concerns, and their information needs. Quantitative data were described, and qualitative thematic analysis of the semi-structured interviews was performed using NVivo data analysis software.

**Results:**

Eighty-six percent of participants indicated they had concerns about their child’s nutrition during treatment. The most common concerns were anorexia, vomiting, and weight loss. While many were happy with the quality of the nutrition support received, one-third of the patients wanted more support. Four key themes emerged from the interviews: (1) patients experience significant and distressing nutrition challenges; (2) patients and families have mixed perceptions of EN; (3) there are gaps in the current nutrition support system for inpatients; and (4) a desire for more accessible nutrition support.

**Conclusion:**

Childhood cancer patients and families experience significant and distressing nutrition challenges during treatment. Standardising information given to patients and their families may optimise nutrition support for paediatric oncology patients and reduce the discordance between families and health professionals. Future implementation of a nutrition decision aid in this population is warranted.

**Supplementary Information:**

The online version contains supplementary material available at 10.1007/s00520-023-07787-3.

## Introduction

A cancer diagnosis and treatment can significantly impact a child’s ability to maintain adequate dietary intake and nutrition [1, 2]. Appetite suppression, vomiting, nausea, mucositis, changes in taste and smell, and pain are side effects of anti-cancer therapies that can negatively affect a child’s nutrition status [3, 4]. Maintaining good nutrition status throughout cancer treatment is crucial for children with cancer [5], as poor dietary intake impacts growth and development, may result in excessive weight gain, decreased tolerance to chemotherapy, and increased risk of infection [6, 7]. Therefore, it is unsurprising that nutrition status is an independent predictor of survival [5].

With many decisions about their child’s treatment being out of their control, feeding their child during treatment is one of the few areas of care parents and families feel they can influence [2, 8]. However, this can significantly strain family relationships and child eating practices. For example, treatment-related weight changes can prompt negative feeding practices such as increasing pressure to eat or compromising diet quality to maintain intake [2, 8]. As parents are highly motivated to seek information about nutrition and health [2, 8], providing reliable and trustworthy nutrition support during treatment is necessary.

Patient and family experiences with nutrition during treatment have been documented [2, 9–13], with most studies investigating patient and parent perceptions of food intake [9, 10, 12] or strategies used by parents to cope with changes in intake [2]. Two studies in Australia and America have explored patient and family perceptions of nutrition support, such as enteral nutrition (EN) and parenteral nutrition (PN) [11, 13]. Given that optimising nutrition status has far-reaching benefits on treatment tolerance, adherence to treatment schedules, and improved quality of life [6, 7], understanding the experience of patients and their families with nutrition as supportive care during treatment is warranted. This study aimed to understand the experience of families caring for a child with cancer in NZ, who have received dietetic support during cancer treatment and their preferences for the delivery, format, and timing of nutrition information.

## Methods

### Setting

This study was conducted at a specialist paediatric oncology centre in Auckland, NZ. Treatment for childhood cancer is coordinated with one other specialist centre, and fourteen shared care centres around NZ. Childhood cancer patients aged 0 to 15 years receiving treatment (which could include chemotherapy, radiation, immunotherapy, or surgery alone or in combination with other modalities) and their families were invited to participate in this study.

### Study design

The study used a mixed methods design with eligible participants identified through discussions with the ward charge nurse and the patient’s assigned nurse. Patients who were classed as palliative, had been recently diagnosed and were not yet ready to receive additional information (at the direction of medical staff), or were deemed too unwell to participate were excluded. The inclusion criteria were otherwise kept broad for patients and their families due to the heterogeneity of this population.

Semi-structured interviews and questionnaire completion was facilitated by one researcher (E.C.) between January and March 2022. The interviewer (E.C) was not involved in the clinical care of participants. Parents or guardians provided written, informed consent before completing the questionnaire and interview. If the patient was older than 6 years, assent was also obtained. The study received ethical approval from the Auckland Health Research Ethics Committee (AHREC) on 20/12/2021 (AH23378). Institutional approval was granted by the ADHB Research Office (Project number: A + 9288).

### Data collection

Participants completed a Health and Nutrition Questionnaire adapted from the literature [14–16] and a semi-structured interview. The questionnaire collected data on the patient’s demographics and diagnosis [17], eating behaviours [18, 19], nutrition education and support received [17], symptom assessment [20], and requirements for nutrition support. The questionnaire was completed via direct entry on an iPad or computer using REDCap electronic data capture hosted by The University of Auckland [21, 22].

The Behavioural Paediatric Feeding Assessment Scale (BPFAS) is a parent-reported questionnaire that gathers information on mealtime behaviours [18]. Only questions related to child eating behaviours were included, which assessed frequencies of behavioural feeding problems and whether parents considered these a problem [18]. Five items from the food responsiveness sub-scale of the Child Eating Behaviour Questionnaire (CEBQ) were also included [19]. These additional questions aimed to capture behaviours of overeating.

The interviews followed a semi-structured interview guide (Supplementary Table 1) and were audio-recorded. Interviews were conducted until thematic saturation on current nutrition support, effectiveness, and requirements for support was achieved. Thematic saturation was determined by repeated themes and an absence of new themes in subsequent interviews. Interview recordings were transcribed verbatim.

### Analysis

Quantitative data were evaluated using SPSS (SPSS Statistics for Windows Version 26, IBM Corp., Armonk, NY, USA), and descriptive statistics reported. Continuous data were presented as mean (SD) and categorical data as frequencies (percentage). Qualitative transcripts were coded line-by-line and analysed using NVivo, 2022, v.12 (QSR International Pty Ltd., Victoria, Australia) using Braun and Clarke’s [23] thematic analysis framework. A multilevel consensus coding methodology was completed to ensure accurate coding and thematic analysis. Seventy-one percent of all interviews (*n* = 15) were coded independently by two investigators (E.C and G.P), who then reviewed their analysis and discussed any discrepancies.

## Results

### Participants

Baseline characteristics are displayed in Table 1. Twenty-one participants completed the Health and Nutrition Questionnaire and a semi-structured interview. Most (48%) were a family member of a child with cancer between the ages of 2 and 4 years. Most (48%) children were of NZ European ethnicity, and one in ten children (9%) were of self-defined Māori ethnicity. Diagnoses were recorded according to ICCC-3 classification [24], with acute lymphoblastic leukaemia (ALL) and Wilms tumour being the most common diagnoses (29% each). Ninety-five percent of children (*n* = 20) were receiving chemotherapy at the time of study participation.

**Table 1:** Participant characteristics and clinical information of family and children with cancer who completed a nutrition questionnaire and semi-structured interviews (*N* = 21) when attending a specialist paediatric oncology centre in New Zealand

Characteristic	Total (%) *n* = 21
Completed questionnaire	
Child with cancer Family member of child with cancer	1 (5) 20 (95)
Age of participant completing questionnaire	
12–15 years 18–30 years 31–40 years 41–50 years	1 (5) 3 (14) 11 (52) 6 (29)
Child age	
< 2 years 2–4 years 4–8 years –12 years 12–16 years	3 (14) 10 (48) 2 (9) 4 (19) 1 (5)
Child main ethnicity	
NZ European Māori Pacific Peoples Asian Middle Eastern/Latin American/ African Other	10 (48) 2 (9) 1 (5) 7 (33) 0 (0) 1 (5)
Child’s diagnosis	
ALL Wilms Neuroblastoma CNS tumour — medulloblastoma Non-Hodgkin’s lymphoma AML Osteosarcoma CNS tumour — glioma Other	6 (29) 6 (29) 4 (19) 0 (0) 2 (9) 3 (14) 3(14) 1 (5) 1 (5)
Current treatment	
Chemotherapy Radiotherapy Surgery: planned Surgery: completed Between treatment cycles Immunotherapy BMT	20 (95) 3 (14) 1 (5) 6 (29) 1 (5) 2 (9) 1 (5)

*ALL*, acute lymphoblastic leukaemia; *AML*, acute myeloid leukaemia; *BMT*, bone marrow transplant; *CNS*, central nervous system

### Health and nutrition questionnaire

Eighteen (86%) participants reported being concerned about their child’s diet or nutrition during treatment (Table 2), with the most common concern being anorexia, or loss of appetite (62%), followed by vomiting (29%), and weight loss (24%). Fourteen (67%) participants rated nutrition as extremely important during childhood cancer; however, only 8 (38%) participants reported nutrition being addressed at every clinic or hospital visit. Over half (52%) of the children had experienced weight loss between 0 and 5 kg since diagnosis, of which 48% (*n* = 10) of parents found worrying.

**Table 2:** Nutrition concerns of family and children with cancer who completed a nutrition questionnaire and semi-structured interviews (*n* = 21) when attending specialist paediatric oncology centre in New Zealand

	Total (%) *n *= 21
Any concerns about child diet or nutrition	
No Yes	3 (14) 18 (86)
Most common concerns	
Anorexia Vomiting Weight loss Meeting nutrient needs NGT dependence/difficulties weaning Oral or food aversions Increased appetite due to steroids	13 (62) 6 (29) 5 (24) 3 (14) 2 (9) 2 (9) 1 (5)
How would you rate the importance of nutrition?	
Extremely important Very important Somewhat important Not so important Not at all important	14 (67) 6 (29) 1 (5) 0 (0) 0 (0)
Nutrition discussed during clinic/hospital visit	
Always Usually Sometimes Rarely Never	8 (38) 10 (48) 1 (5) 1 (5) 1 (5)
Weighed for day stay or inpatient treatment	
Always Usually Sometimes Rarely Never	17 (81) 4 (19) 0 (0) 0 (0) 0 (0)
Weight loss since diagnosis	
No: weight hasn’t changed No: weight has increased Unsure Yes	5 (24) 5 (24) 0 (0) 11 (52)
Weight loss since diagnosis	
0–5 kg > 5 kg	8 (38) 3 (14)
How does your child’s weight loss make you feel?	
Not worried Don’t mind Worried Very worried	0 (0) 1 (9) 6 (55) 4 (36)
Noticed muscle loss	
Yes — a lot Yes — a little No change Unsure	7 (33) 5 (24) 7 (33) 2 (9)

*NGT*, nasogastric tube

Most (67%) participants reported changes in their child’s diet following diagnosis (Supplementary Table 2). Common dietary changes included a focus on food safety (29%), deterioration in intake (21%), and an impact on the types and amounts of foods accepted (21%). All participants (100%) had received nutrition care or advice from the hospital dietitian, and seven (33%) reported receiving nutrition information from their nurse specialist. The types of advice provided by their health care team focused on food safety (62%), general healthy eating for cancer (62%), and how to gain weight (24%). Most participants (85%) rated the nutrition advice from the dietitian as helpful (ranging from somewhat, very, or extremely helpful) and that the dietitians were responsive to their queries. Reasons for rating advice from the dietitian as ‘somewhat helpful’ included a resulting lack of knowledge on appropriate low-risk foods, that the focus appeared to be on weight maintenance rather than diet quality, and that they were only contacted when nasogastric (NG) feeding was recommended.

One-third (*n* = 7) of participants reported that they would have liked to have received more support from their dietitian when their child was on treatment. Suggestions for further support included online weekend support, meal plans/family meal ideas when at home, a list of ‘safe’ foods, early support to prevent oral aversions and poor eating behaviours, and general lifestyle advice.

The Memorial Symptom Assessment Scale (MSAS) [25] was mostly parent-completed (Table 3). The mean (SD) number of symptoms experienced was 6.3 (4.2). Symptom prevalence in the last week experienced by most children included as follows: lack of energy (71%), pain (62%), lack of appetite (62%), diarrhoea (57%), nausea (57%), vomiting (48%), irritability (48%). Of these, symptoms such as lack of appetite, nausea, and vomiting directly impact nutrition. Other symptoms reported as less frequent within the last week but can indirectly impact nutrition include anxiety, depression, or pain impacting desire to eat or low energy/stamina to complete meals. Over half (55%) of the symptoms in the last week were rated moderate to very severe in all children who experienced them. Weight loss and constipation caused high levels of distress and severity, whereas nausea, vomiting, and lack of appetite were more likely to be rated as severe than distressing.

**Table 3:** Parent-completed symptom assessment using the Memorial Symptom Assessment Scale (MSAS) [21] conducted when attending specialist paediatric oncology centre in New Zealand (*n* = 21)

Symptom	Prevalence (%)			
	Overall, *n* (%)	Severity: Mod-V severe, *n* (%)^a^	Frequency: a lot — almost always, *n* (%)^b^	Distress: quite a bit — very much, *n* (%)^c^
Lack of energy	15 (71)	14 (93)	3 (20)	4 (27)
Lack of appetite	13 (62)	13 (100)	3 (23)	4 (31)
Pain	13 (62)	13 (100)	2 (15)	6 (46)
Nausea or feeling like you could vomit	12 (57)	11 (92)	2 (17)	5 (42)
Diarrhoea or loose bowel movements	12 (57)	12 (100)	3 (25)	4 (33)
Vomiting	10 (48)	9 (90)	3 (30)	5 (50)
Feelings of being irritable	10 (48)	9 (90)	2 (20)	2 (20)
Hair loss	10 (48)	9 (90)	-	4 (40)
Feelings of sadness	9 (43)	8 (89)	1 (11)	2 (22)
Feeling of being drowsy	9 (43)	9 (100)	2 (22)	2 (22)
Feeling of being nervous	8 (38)	8 (100)	0 (0)	4 (50)
Worrying	7 (33)	5 (71)	0 (0)	1 (14)
Change in the way food tastes	7 (33)	3 (43)	-	0 (0)
Skin changes	7 (33)	7 (100)	-	1 (14)
Dry mouth	6 (29)	5 (83)	0 (0)	0 (0)
Weight loss	5 (24)	4 (80)	-	3 (60)
Itching	4 (19)	4 (100)	1 (25)	2 (50)
Mouth sores	4 (19)	4 (100)	-	2 (50)
Difficulty sleeping	4 (19)	4 (100)	1 (25)	2 (50)
Dizziness	3 (14)	3 (100)	0 (0)	1 (33)
Difficulty swallowing	3 (14)	3 (100)	0 (0)	0 (0)
Constipation	3 (14)	3 (100)	-	2 (67)
Numbness/tingling	2 (10)	2 (100)	1 (50)	0 (0)
Problems with urinating	2 (10)	1 (50)	0 (0)	0 (0)
Difficulty concentrating or paying attention	1 (5)	1 (100)	1 (100)	0 (0)
Cough	1 (5)	0 (0)	0 (0)	0 (0)
Shortness of breath	1 (5)	1 (100)	0 (0)	0 (0)
Swelling in arms or legs	1 (5)	1 (100)	-	0 (0)
‘I don’t look like myself’	0 (0)	0 (0)	-	0 (0)

^a^Number (%) of patient rated as moderate to very severe intensity

^b^Number (%) of patient rated as a lot to almost always frequency

^c^Number (%) of patient rated as quite a bit to very much distress

### Semi-structured interviews

Twenty-one (*n* = 21) semi-structured interviews were conducted with childhood cancer patients and members of their families in person (*n* = 19) or via telephone (*n* = 2). These included three patients and twenty-three family members. The emerging themes were organised into four categories: (1) patients experience significant and distressing nutrition challenges; (2) patients and families have mixed perceptions of EN; (3) there are gaps in the current nutrition support system for inpatients; and (4) a desire for more accessible nutrition support. Sub-categories for these themes and representative quotes are outlined in Table 4.

**Table 4:** Emergent themes from semi-structured interviews with childhood cancer patients and family members (*n* = 21) on their needs for nutrition support and dietary management during treatment conducted when attending specialist paediatric oncology centre in New Zealand

	Number of respondents (*N* = 21)	Quote
Theme 1: Patients experience significant and distressing nutrition challenges		
Families experience frustration and distress associated with weight and dietary changes		
Frustration and distress associated with weight and dietary changes	9	*Yeah, I am actually anxious like, you know, when it comes to mealtime because he doesn’t have – he doesn’t have an appetite.* — Interview 5
Weight loss challenges/accepting weight loss as inevitable	14	*Cause he was like 38 kilos when we came in in September last year, and then by the time we went – left to go home the first time we could go home, he’d lost probably I think he went down to nearly 31 kilos. So he had lost quite a bit of weight, so I’m quite glad that he came in with a bit of weight behind him* — Interview 1
Managing changing tastes	5	*Their tastebuds change really quickly so, that’s the hardest part is that she’ll really like something one week and the next week she’s like “No. Absolutely not.”* — Interview 19
Struggle to communicate with child	6	*Like a three year old has all the will in the world but it – it’s often quite difficult to explain about food and the importance for nutrition.* — Interview 21
Struggling with nutrition at home	6	*It was just completely draining and depressing spending my whole days trying to feed her through the NG and she’d just vomit it all up and not gain any weight.* — Interview 18
Fussy eating is common and creates negative feeding practices		
Fussy eating challenges	-	
*Child missing milestones*	5	*We were about to start doing solids at home because she was, you know, almost coming up 6 months and showing signs of being interested but then we came in and it just hasn’t happened.* — Interview 10
*Food aversions*	15	*Yeah, I’d kind of eat one food, throw up and I would just – I couldn’t even think about the food, let alone like look at it.* — Interview 9
*Periods of inadequate intake*	9	*When he first got diagnosed and started chemo, he didn’t eat for about nine days and he was vomiting a lot, like a lot during every day.* — Interview 1
*Poor diet quality*	10	*I would say sugar’s definitely one of the, you know, convenience food that’s easy to – or I’m making things sweeter just to keep him more interested.* — Interview 13
Negative feeding interactions	-	
*Child dictates diet*	4	*We have found we’ve tried to indulge her whims to like she will – she’ll randomly say “oh, I really feel like this.” And ‘cause you’re so conscious of trying to get food into her you’re like “okay!”* — Interview 19
*Food as a reward*	2	*But then you also need to reward these kids after treatment, so you just give them what they really want during that short period of time.* — Interview 4
*Pressure to eat*	3	*there’s a lot of pressure on you to try and force them to eat, you know […] And you’re trying to sort of get this food into them and fatten them up or whatever.* — Interview 19
*Prioritising intake over diet quality*	13	*Usually [healthy eating is] quite important to me. But, right now it’s like, he’s alive. And we’re just trying to keep him from losing weight so the fruits and veggies can, you know, be put on hold, unless he feels like it.* — Interview 8
Theme 2: Patients and Whānau have mixed perceptions of EN		
Challenges of EN	-	
Distress of NGT issues	7	*I mean it’s quite a traumatic thing to have [the NGT] put in anyway and then you throw it up again. It’s – it’s really quite traumatic.* — Interview 20
Fear of reliance on tube feeds	4	*While we can use the tube, I don’t want to be relying on it when we don’t necessarily have to. I’d like her to keep up her, you know natural feeding where we can* — Interview 10
Perception that EN disrupt oral intake	7	*I don’t want to intervene [with a NGT] too soon to the point where his stomach’s full and he doesn’t feel hungry so he doesn’t want to eat.* — Interview 13
Delaying EN initiation	5	*I knew we were probably gonna reach [inserting an NGT] at a point but I was trying to stall it for as long as I could.* — Interview 19
Positive view of EN	9	*I think [the NGT] shouldn’t be suggested, I think it should be informed that you have to have it […]yeah, no its more reassuring to know that he’s getting intake* — Interview 3
Theme 3: There are gaps in the current nutrition support system		
Limited contact with dietitians		
Dietitian contact often responsive to weight issues	6	*So, I think the name of the game up here is more so to make sure the kids are maintaining their weight just to make it through the chemo and that food or diet, as long as their eating, doesn’t seem to be as big of thing.* — Interview 13
Difficulty accessing dietitian	2	*When you’re only coming in once a week to clinic and you’re hoping, I guess, you’re gonna see a dietitian then. I don’t know whether you’re guaranteed that you will.* — Interview 21
Unreliable sources of information	4	*I actually had a guy that I bumped into that […] believes in healing through nutrition. […] So, he did give me some sheets which were quite interesting that just said things that can—which [were] actually quite good to support [him].* — Interview 13
Barriers to implementing dietary advice		
Limited food options from kitchen	4	*Not to be rude, but I don’t really think the food here is really appetising, especially for kids. The taste is really bland. That’s why maybe they don’t eat properly.* — Interview 7
Overwhelmed at diagnosis so struggle to take in information	8	*Parents get sometimes – get really overwhelmed in diagnosis days, they cannot process information with those things.* — Interview 4
Parents need to piece together information from multiple health professionals	3	*I’ve sort of got bits here and there and I sort of can join it all together and I’ve sort of discussed “oh, well this and these guys said this and so forth.”* — Interview 10
Treatment side effects impacting intake	16	*He’s getting just over half of his daily* – *what he needs, but then if we put that up, he’s just gonna spew up because he can’t handle it, so it’s like well. It’s like a…just a balancing act between throwing up and him keeping it down.* — Interview 8
Delaying addressing issues until treatment completion	6	*He’s got three months left of chemo and then he should be finished completely. So, I’m hoping that once this finishes and then he might come back to his old self.* — Interview 17
Little to no use of alternative medicines		
Families don’t want to use alternative medicine	7	*I guess I’m very conscious that that’s a risky business with a blood cancer so I’m not sure that we will to be honest […] The reality is almost certainly I won’t use anything* — Interview 21
Open to alternative medicine use but will be cautious of any unintended consequences	6	*We do [want to use alternative medicines] but we usually run it past the doctors because we do have trust in them and a lot of what [my child] is going through is well out of our depth.* — Interview 13
Theme 4: Desire for more accessible nutrition support		
General support accessible at any time		
Pamphlet or online resources are accessible at any time	7	*I found that pamphlets were quite good. ‘Cause the thing is – oh, even online as well ‘cause we’re here at the hospital. There’s a lot of down time here.* — Interview 15
More information around healthy eating	4	*But I just wasn’t like told about like healthy food […] I was never told about like – is she supposed to be eating like fruits and vegetables and all that stuff or is she supposed to avoid [them].* — Interview 2
Recipes/meal ideas	3	*And maybe recipes, more recipes. Just easy ones, ‘cause you know, when your brain’s not working, you can’t think of…I was just heating up tinned spaghetti.* — Interview 14
More access to a dietitian for personalised support		
Constructive strategies to implement change	4	*Maybe help getting her to have some more solids in the future, if someone had any tips on that kind of thing.* — Interview 18
More regular dietetic support	2	*‘Cause like they’re “okay, so [this is] the information that he needs.” Okay, fair enough. You take the information, you process it, then afterwards you don’t get to see [the dietitian] for another what? Three months.* — Interview 4
Want personalised support	9	*I mean the problem with written or online resources – which they are great – is if you’ve got a question about your particular scenario, you’ve gotta have someone else to ask ‘cause it might not be covered in that information.* — Interview 21
Support throughout treatment		
Support starting at diagnosis	4	*No, I think [support] was good in the beginning ‘cause it kind of prepared me when I went home.* — Interview 14
Support pre-emptive of challenges	3	*Maybe, you know, once she’s hit a really good weight and we’re like, you know, anticipating that the tube will be out within the next six months or whatever then I’d want to be – probably want some support in getting her to take solids a bit more.* — Interview 18
Support on treatment completion	2	*That would be really good to – on discharge to have some of those options because that’s when you can use them because he’s recovering.* — Interview 13
Receiving adequate support		
Appreciation for nutrition support received	13	*So basically from the moment we’ve come up we’ve received support. So yeah, it’s been good, and it’s been helpful.* — Interview 10

#### Theme 1: Patients experience significant and distressing nutrition challenges

Most patients experienced challenges related to limited dietary intake or fussy eating, which caused frustration and distress. Managing taste changes, difficulties communicating with young children, and accepting that weight loss was inevitable were all significant challenges during treatment.

*Yeah, I’d kind of eat one food, throw up and I would just – I couldn’t even think about the food, let alone like look at it. So, for a little bit I went off potatoes*. — Interview 9 (CCS: male, osteosarcoma, 12–15 years)

Fussy eating encompassed food aversions, periods of inadequate intake, poor diet quality, and missed feeding milestones. While some families described being able to cope with these challenges and viewed it as a phase, other families described how it significantly impacted their child’s healthy eating practices.

*Usually [healthy eating is] quite important to me. But, right now it’s like, he’s alive. And we’re just trying to keep him from losing weight so the fruits and veggies can, you know, be put on hold, unless he feels like it.* — Interview 8 (Mother: male, Wilms tumour, 2–4 years)

#### Theme 2: Patients and families have mixed perceptions of enteral nutrition

Placement of NG tubes for feeding was common, with families having a mixed view of their benefits. Some parents were positive about the role of NG feeds for maintaining weight and providing optimal nutrition. However, initiating NG feeding was often communicated to parents as a suggestion rather than an instruction or part of the treatment protocol, leading to confusion about their necessity.

*I think [the NGT] shouldn’t be suggested; I think it should be informed that you have to have it […] yeah, no, it’s more reassuring to know that he’s getting intake*. — Interview 3 (Father: male, ALL, 2–4 years)

Reasons for hesitancy included fear of complications, developing a reliance on EN, and disruption to oral intake. Two families delayed NG insertion as they felt they had not received enough explanation of the mechanism of feeding, the benefits of EN, and the proposed duration of use.

*I knew we were probably gonna reach [inserting an NG tube] at a point, but I was trying to stall it for as long as I could.* — Interview 19 (Mother: female, osteosarcoma, 12–15 years)

*We need to be guided by, yes, okay, cool, we’re gonna be on the tube. She’ll need that for probably a week. And then she’ll probably go back to feeding…… And then, this is how we introduce solids into the mix as well,” is sort of, you know, how I would imagine that would sort of get – get there*. — Interview 10 (Mother: female, CNS tumour, < 2 years)

#### Theme 3: There are gaps in the current nutrition support system

Some patients and families reported limited contact with the dietitian. Dietitians on the wards often need to prioritise patients with significant weight loss or intensive nutrition support, e.g. PN. Families expressed difficulties accessing dietitians for other concerns and often sought alternative sources of information. If they could see a dietitian, participants reported significant barriers to implementing nutrition advice, such as treatment side effects impacting intake.

*When you’re only coming in once a week to clinic and you’re hoping, I guess, you’re gonna see a dietitian then. I don’t know whether you’re guaranteed that you will. *— Interview 21 (Mother: female, ALL, 2–4 years)

There was a consensus that there was information overload at diagnosis, with limited capacity to absorb information beyond diagnosis and treatment protocols.

*Parents get sometimes get really overwhelmed in ‘diagnosis days.’ They cannot process information*. — Interview 4 (Father: male, neuroblastoma, 9–12 years)

Parent desire to delay addressing nutrition until after treatment had finished was apparent, as was the limited food availability in/near the hospital.

*He’s got three months left of chemo and then he should be finished completely. So, I’m hoping that once this finishes and then he might come back to his old self.* — Interview 17 (Mother; male, Lymphoma, 5–8 years)

Parents reported hesitation when asked about alternative or homoeopathic nutrition approaches. Most parents did not report using any natural remedies, but when they did, there was always a certain level of care and caution taken to ensure that it did not interact with any of the therapies they were receiving and have unintended consequences.

*Instead of using like the paraffin creams all the time, we asked if maybe we could use some manuka honey. […] But again, the doctors – with his immunity dropping so low, any bacteria sometimes can be really harmful.* — Interview 13 (Mother: male, AML, 2–4 years)

*My husband found that [manioc] has like B19 or something so it’s like an antioxidant, yeah. And it’s, like you know, it’s a traditional breakfast we eat it so […] those things which—which are like sort of highlighted, we would incorporate it more. *— Interview 5 (Mother: male, neuroblastoma, 2–4 years)

Other parents were more confident and would openly disclose natural remedies they had used;

*When it comes to her vomiting, […] I’ll try give her like gingernuts or something like that or make sure there’s like ginger like in our food. *— Interview 16 (Mother: female, neuroblastoma, 2–4 years)

#### Theme 4: Desire for more accessible nutrition support

Finally, while some families felt they were receiving adequate support, many wanted more accessible nutrition support. Easily accessible, general nutrition support (e.g. healthy eating and nutritious meal ideas) that could be accessed when patients or caregivers had the time and headspace were requested.

*I found that pamphlets were quite good. ‘Cause the thing is – oh, even online as well ‘cause we’re here at the hospital. There’s a lot of downtime here.* — Interview 15 (Mother: female, Burkitt’s lymphoma, 2–4 years)

Others requested increased access to dietitians for more personalised support. For example, guidance on making up for missed feeding milestones, or support for healthy eating while their child was immunocompromised. In terms of timing, preferences varied and included support at diagnosis, and on treatment completion. Some families wanted support to be available pre-emptive of nutrition challenges.

*I mean the problem with written or online resources […] is, if you’ve got a question about your particular scenario, you’ve gotta have someone else to ask ‘cause it might not be covered in that information.* — Interview 21 (Mother: female, ALL, 2–4 years)

### Relationship between emergent themes

**Fig. 1:**
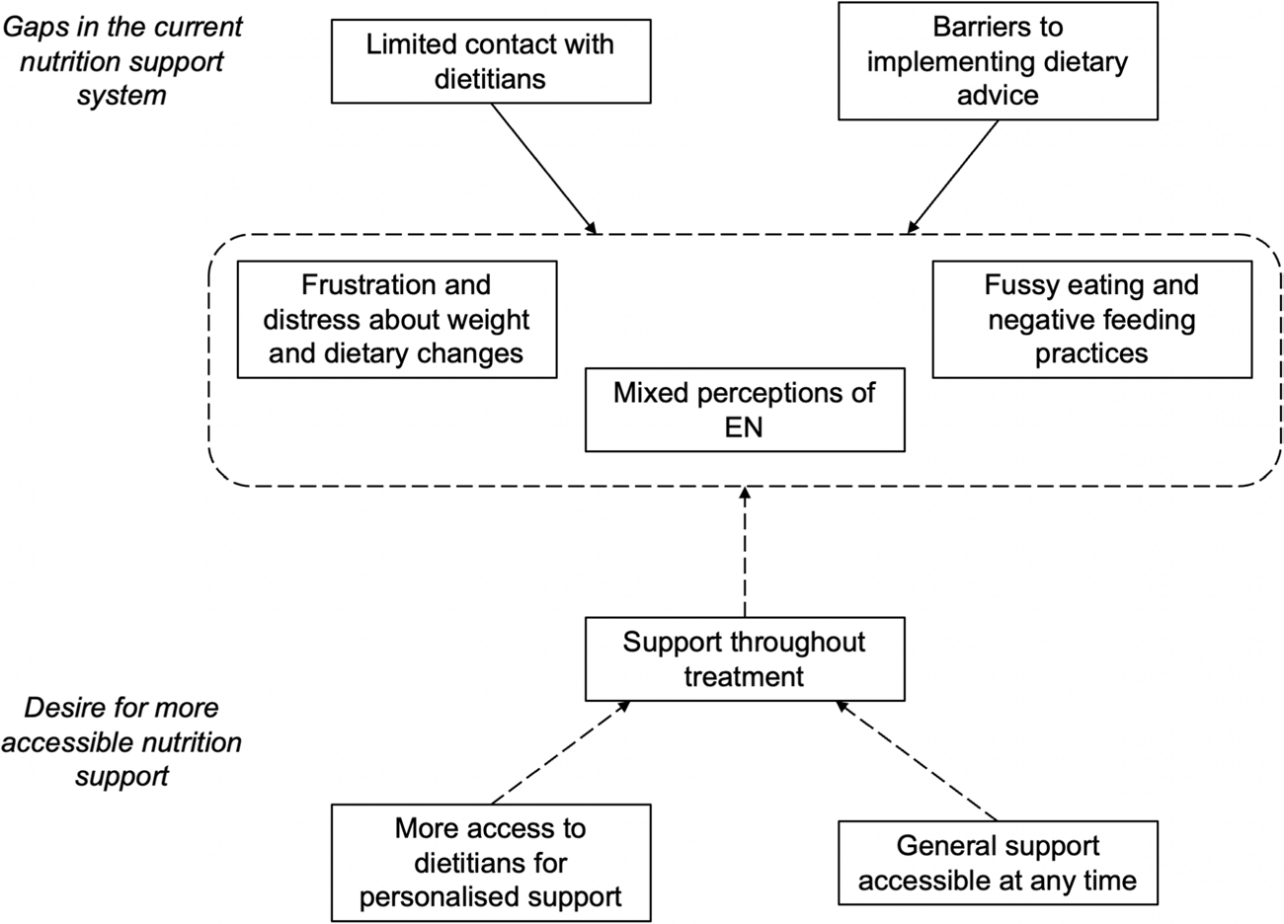
Concept map of emergent themes from interviews with childhood cancer patients and their families on their experiences with nutrition support while undergoing treatment

A concept map is displayed in Fig. 1 to illustrate the relationship between the emergent themes. The current gaps in nutrition support that may contribute to the challenges faced by childhood cancer patients and their families were identified. Stretched dietetic resource and existing barriers to implementing dietary advice create an environment where nutrition support is often deescalated during treatment. This may create feelings of isolation when faced with challenges such as fussy eating/food aversions, feelings of frustration and distress, and mixed perceptions about the benefits of EN.

Some families were very happy with the current dietary support provided. However, requests for more readily available additional support throughout their cancer treatment journey were made. Increased access to the hospital dietitian is required for more personalised, patient-centred support. Additionally, pamphlets and online resources providing general nutrition support were useful for patients and families to access in their own time.

## Discussion

This study aimed to understand the experience of families caring for a child with cancer in NZ, who received nutrition and dietetic support during cancer treatment and determine preferences for the delivery, format, and timing of nutrition information/support. Nutrition-related challenges during treatment included fussy eating, weight changes, and mixed perceptions of EN, which families found distressing. These challenges were often a result of the intensive cancer therapies; however, perceived limitations to accessing ward dietitians and existing barriers to implementing nutrition advice may exacerbate these experiences.

Two prevalent nutrition-related challenges resulting in feelings of frustration and distress were changes in weight and fussiness. Over half of the children had experienced weight loss since diagnosis, and almost a quarter of parents reported an impact on the types and amounts of foods accepted by their child. Food aversions, inadequate intake, poor diet quality, and missing feeding milestones were all reported by families and were categorised as ‘fussy eating challenges’. These challenges have previously been reported in the literature [2, 8, 10, 26] alongside malnutrition [5, 6] and are often intertwined [9]. Lower BMI percentile at diagnosis and during treatment are related to greater parent-reported resistance to eating and aversions to mealtimes [9].

The contribution of treatment side effects to overall intake exacerbated parent stress, with an average of 6.3 symptoms known to disrupt oral intake reported [10]. These sentiments are echoed in cohorts around the world, with Swedish parents reporting the responsibility of getting their child to eat as distressing, often resulting in coercive feeding practices to increase intake [12]. Negative feeding practices, such as coercion, were also commonly discussed in this study. Families used strategies to prioritise total food intake over diet quality, pressuring their child to eat or allowing their child to dictate their food choices. Similar findings were reported in Australia, where parents reported feeling stressed and engaging in conflict with their child at mealtimes, often turning to the use of pressure, food bribes, or threats of an NG tube to coerce their child into eating [2].

Perceptions of EN, such as NG feeding, were mixed. Some families had faced mechanical challenges (e.g. tube expulsion or blocking), which contributed to negative perceptions. Other reasons for resistance to EN included concerns about becoming reliant on this form of nutrition or that EN would disrupt oral intake and the body’s ‘natural’ hunger and fullness sensations. Often it was felt that health professionals did not adequately address these questions and concerns. The use of EN during childhood cancer treatment is common, particularly in the prevention of malnutrition [27, 28]. However, inconsistent recommendations from health care professionals may contribute to families’ uncertainty around its benefit [13, 29]. Cohorts of parents in Australia [13] and America [11] have demonstrated similar conclusions, with 100% of parents interviewed in an American cohort preferring the use of PN if their child was unable to eat, despite EN being the safer method [11]. Both studies concluded that standardised, evidence-based information from health professionals was essential for patients and families to make informed decisions [11, 13]. This was also apparent in the present study, where one caregiver reported delaying NG tube insertion because they believed they would have to prepare and blend up meals to use as the feed. Standardised information and nutrition pathways may minimise delays to appropriate nutrition support and reduce malnutrition risk.

Current guidelines for best practice reinforce the importance of early and sustained dietitian involvement in patient care [28, 30, 31]. Families perceived limited access to the ward dietitian as a potential risk factor for increased nutrition challenges. All participants had contact with a dietitian, and the quality of the consults was reported to be strong. However, there was a need for increased frequency of support outside of weight loss or EN. Modifying practice to a more proactive approach could reduce the risk of significant malnutrition in this patient group [28, 30].

Feeling stressed or overwhelmed while receiving large amounts of new information has been shown to impact families’ recall [13, 32]. Poor retention of information has previously been identified, with > 50% of parents reporting little to no recall of the first two consults with their child’s oncologist [32]. Despite the importance of adequate nutrition in a patient’s treatment plan, decision-making around initiating nutrition support is difficult for parents and healthcare professionals. Shared decision-making (SDM) is the best practice in patient-centred care, where treatment decisions are made in collaboration with the patient (their family) and health professional [33]. Decision aids (DA) are one way to implement SDM in clinical practice by providing patients with evidence-based options within the context of their preferences and values [34]. Using a DA for nutrition support following a childhood cancer diagnosis would guide patients through a deliberative process of actively weighing up the requirements for nutrition with the pros and cons of engaging in nutrition support during treatment. Nutrition DA’s have been piloted in Australian paediatric populations [35, 36] and warrant implementation in NZ.

This study validates findings from international studies [2, 9–13] and provides an NZ context to the priorities and needs of patients and families. Hearing perspectives from Māori, Pacific, and Asian families reflects the diverse population in NZ. However, the participation of Māori (9%) and Pacific (5%) families was low compared to national averages [37]. Sampling bias must be considered, as participation was voluntary and during a defined period; therefore, families with particularly distressing experiences with nutrition may have been more likely to participate. Excluding patients who were too unwell to participate (at the discretion of the medical team) may have led to the exclusion of patients and their families most at risk of malnutrition. The distribution of diagnoses was not reflective of the annual distribution, where the most common cancer diagnosis in New Zealand in 2021 were CNS tumours (24%), then leukaemias (23%), and lymphomas (17%) [37]. The limited data collection timeframe may have influenced this, as would the diagnoses admitted to the ward, which were majority ALL and solid tumours such as Wilm’s tumours or neuroblastomas. Additionally, this study may have limited generalisability due to its focus on NZ experiences.

## Conclusion

Childhood cancer patients and families experience significant and distressing nutrition challenges during treatment, including fussy eating, weight fluctuations, and mixed attitudes towards EN. Despite the importance of nutrition support in a patient’s treatment plan, decision-making around its initiation is difficult for parents and healthcare professionals. Standardising information given to patients and their families through a DA may optimise nutrition support for paediatric oncology patients and reduce the discordance between families and health professionals. Future implementation of a nutrition DA in this population is warranted.

## Supplementary Information

Below is the link to the electronic supplementary material.

Supplementary file1 (DOCX 21 KB)

## Data Availability

The data supporting this study's findings are available from the corresponding author, ALL, upon reasonable request.
