# Supplementary material for: Understanding the patient and family experience of nutrition and dietetic support during childhood cancer treatment
Source: Support Care Cancer. 2023 May 8;31(6):326. doi: 10.1007/s00520-023-07787-3 (PMC10167176; doi:10.1007/s00520-023-07787-3)
Supplement: Supplementary file 1 — Supplementary file1 (DOCX 21 KB) [file 520_2023_7787_MOESM1_ESM.docx]

**Supplementary Table 1.** Semi-structured guide for interviews with childhood cancer patients and family members on their needs for nutrition support and dietary management during treatment

|  | **Core Questions** | **Probes** |
| --- | --- | --- |
|  | **Introduction** | |
| Participant introduction | “Can you please tell us your name, age, your cancer diagnosis and what treatments you are currently or have previously received’ |  |
|  | **Experiences Navigating Nutrition** | |
| Nutrition | What kinds of support have you received or sought out regarding the nutrition and diet of your child? | - Diet - Lifestyle - Physical activity - Food safety |
|  | Who has provided this support? | Probe where this support was accessed i.e. through the DHB, privately or Google |
|  | What type of challenges have you/your child faced with regards to nutrition since your diagnosis? | - Nausea/vomiting - Diarrhoea - Constipation - Loss of appetite - Mouth pain |
|  | What challenges have you faced when you have tried to keep up your normal eating and drinking? | - Pain - Pressure - Anxiety |
| Sources of information | Where have you gone to receive nutrition information when on treatment? | - Hospital dietitian - Private dietitian/nutritionist - Google - Good/bad experience? |
|  | What types of challenges have you faced in accessing and understanding this information? | - Lack of time - Lack of support - Health |
|  | What concerns do you have regarding your child’s current diet? |  |
|  | What challenges do you face in trying to pursue healthy eating habits with your child? |  |
| Delivery | What kinds of additional support would you find beneficial? | Why? |
|  | Who would you want to talk about this information with? |  |
|  | How do you think this information should be given out? | - Social worker - Nurse - Doctor - Dietitian - Parent - Friend |
|  | What would your ideal platform be for this nutrition support? | - Online - Paper - App - Groups   Why?  Probe on which platform would be most helpful and accessible |
|  | What would be the best time for this support to start? | Why? |
|  | How should this information look? | - Small bitesize pieces - Lists - Facts - Instructions - Pictures |
| Traditional and complementary medicine | During your child’s treatment have you used or thought about using any traditional or alternative medicines, Rongoā Māori or rākau (plant remedies)? |  |
|  | What were these? |  |
|  | Have you ever, or have you ever wanted to discuss traditional or alternative medicines, Rongoā Māori or rākau (plant remedies) with your child’s medical team |  |
|  | If you have chosen to use traditional or alternative medicines, Rongoā Māori or rākau (plant remedies), what were the reasons for their inclusion? |  |
| Final question | Finally, is there anything about nutrition challenges and support that has not been discussed that you feel strongly about and would like to bring up now? |  |
|  | **Closing** | |
| Conclusion | Thank you. Your answers and discussion have been very helpful and informative. We are very grateful for the information that you have provided. | |

**Supplementary Table 2.** Nutrition information and support received by family and children with cancer who completed a nutrition questionnaire and semi-structured interviews (*n* = 21) when attending specialist paediatric oncology centre in New Zealand.

|  | **Total (%)**  ***n* = 21** |
| --- | --- |
| Changed child’s diet after diagnosis  No  Yes | 7 (33)  14 (67) |
| Types of changes  Types and amounts of foods  More fresh food/less leftovers  Introduced nutrition support (oral/enteral)  Low risk foods  Deterioration in intake (energy dense foods) | 3 (21)  3 (21)  3 (21)  4 (29)  3 (21) |
| Belief that children with cancer require specific food(s)  No  Yes | 5 (24)  16 (76) |
| Types of specific foods  High energy  Sugar avoidance  High protein  Low risk  Fruits and vegetables  Salty foods | 2 (13)  1 (8)  3 (19)  2 (17)  7 (44)  2 (13) |
| Think certain foods increase white blood cell counts  No  Yes | 18 (86)  3 (14) |
| Avoided any foods since diagnosis  No  Yes | 11 (52)  10 (48) |
| Types of advice provided by health care team  General healthy eating  Specific foods to avoid  Specific foods to eat  Portion sizes  How to lose weight  How to gain weight  Recipes  Vitamin and mineral supplements  Protein and energy supplements  Other supplements  Physical activity/exercise  Where to find advice online  Nutrition support groups  Using diet to manage symptoms  Soft/liquid diets  Food safety  None of the above  Other | 13 (62)  11 (52)  7 (33)  4 (19)  0 (0)  5 (24)  3 (14)  1 (5)  4 (19)  3 (14)  3 (14)  4 (19)  1 (5)  2 (9)  2 (9)  13 (62)  6 (29)  2 (9) |
| Received nutrition care/advice from:  Hospital dietitian  Community dietitian  Nurse  Doctor  Private nutritionist  Other | 21 (100)  0 (0)  7 (33)  10 (28)  0 (0)  1 (5) |
| Rating nutrition care advice from dietitian  N/A, I did not receive any advice from a dietitian  Extremely helpful  Very helpful  Somewhat helpful  Not so helpful  Not at all helpful | 0 (0)  7 (33)  8 (28)  5 (24)  1 (5)  0 (0) |
| Other support would have liked to receive  I received enough support  I would have liked more support | 14 (67)  7 (33) |
| Other searches for information or advice on diets  No  Yes | 14 (67)  7 (33) |
| Where advice was accessed  Online  Booklets provided by hospital  Parents of children with cancer | 6 (29)  2 (9)  1 (5) |
